# The Immunoregulatory Role of the Signal Regulatory Protein Family and CD47 Signaling Pathway in Type 1 Diabetes

**DOI:** 10.3389/fimmu.2021.739048

**Published:** 2021-09-16

**Authors:** Robert C. Sharp, Matthew E. Brown, Melanie R. Shapiro, Amanda L. Posgai, Todd M. Brusko

**Affiliations:** ^1^Department of Pathology, Immunology, and Laboratory Medicine, College of Medicine, University of Florida, Gainesville, FL, United States; ^2^Department of Pediatrics, College of Medicine, Diabetes Institute, University of Florida, Gainesville, FL, United States

**Keywords:** CD47, SIRPG, SIRPA, SIRPB1, type 1 diabetes, signal regulatory protein

## Abstract

**Background:**

The pathogenesis of type 1 diabetes (T1D) involves complex genetic susceptibility that impacts pathways regulating host immunity and the target of autoimmune attack, insulin-producing pancreatic β-cells. Interactions between risk variants and environmental factors result in significant heterogeneity in clinical presentation among those who develop T1D. Although genetic risk is dominated by the human leukocyte antigen (HLA) class II and insulin (*INS*) gene loci, nearly 150 additional risk variants are significantly associated with the disease, including polymorphisms in immune checkpoint molecules, such as *SIRPG*.

**Scope of Review:**

In this review, we summarize the literature related to the T1D-associated risk variants in *SIRPG*, which include a protein-coding variant (rs6043409, G>A; A263V) and an intronic polymorphism (rs2281808, C>T), and their potential impacts on the immunoregulatory signal regulatory protein (SIRP) family:CD47 signaling axis. We discuss how dysregulated expression or function of SIRPs and CD47 in antigen-presenting cells (APCs), T cells, natural killer (NK) cells, and pancreatic β-cells could potentially promote T1D development.

**Major Conclusions:**

We propose a hypothesis, supported by emerging genetic and functional immune studies, which states a loss of proper SIRP:CD47 signaling may result in increased lymphocyte activation and cytotoxicity and enhanced β-cell destruction. Thus, we present several novel therapeutic strategies for modulation of SIRPs and CD47 to intervene in T1D.

## Introduction

Type 1 diabetes (T1D) pathogenesis involves marked failures in immunoregulation and an adaptive immune response targeting β-cell autoantigens expressed in the pancreatic islets of Langerhans. Genome-wide association studies (GWAS) have shown that T1D is a highly polygenic disease (1–3). The majority of T1D risk is conferred by the highly polymorphic human leukocyte antigen (HLA) class II region and the insulin locus; however, there are nearly 150 additional single nucleotide polymorphisms (SNPs) associated with T1D risk (1, 3, 4). A subset of these SNPs impact CD4^+^ and CD8^+^ T cell function, including risk variants tagged to co-stimulatory and co-inhibitory molecules *CD226, CTLA4*, and *SIRPG* (2, 3).

Among these variants, those associated with *SIRPG* (signal regulatory protein gamma), which encodes the receptor-like transmembrane protein SIRPγ, have been proposed to modulate T cell and natural killer (NK) cell activation (5–7). *SIRPG* contains two SNPs associated with risk for T1D (5, 8–12): rs2281808 [C>T, intronic, minor allele frequency (MAF): 0.27, odds ratio (OR): 1.11] and rs6043409 (G>A, Ala263Val, MAF: 0.20, OR 1.13). These SNPs are in tight linkage disequilibrium (LD) (*R^2^ = 0.94; D’ = 0.98*) (8, 10, 13–15) and tend to be inherited as a haplotype that carries either risk (C/G, 65.2%) or protection (T/A, 33.5%) from T1D in European cohorts (13). In addition to T1D, rs2281808 and rs6043409 are associated with other T cell-mediated autoimmune diseases, such as rheumatoid arthritis (RA), systemic lupus erythematosus (SLE), and ulcerative colitis (UC) (6–8, 10, 14–17).

**Figure 1:**
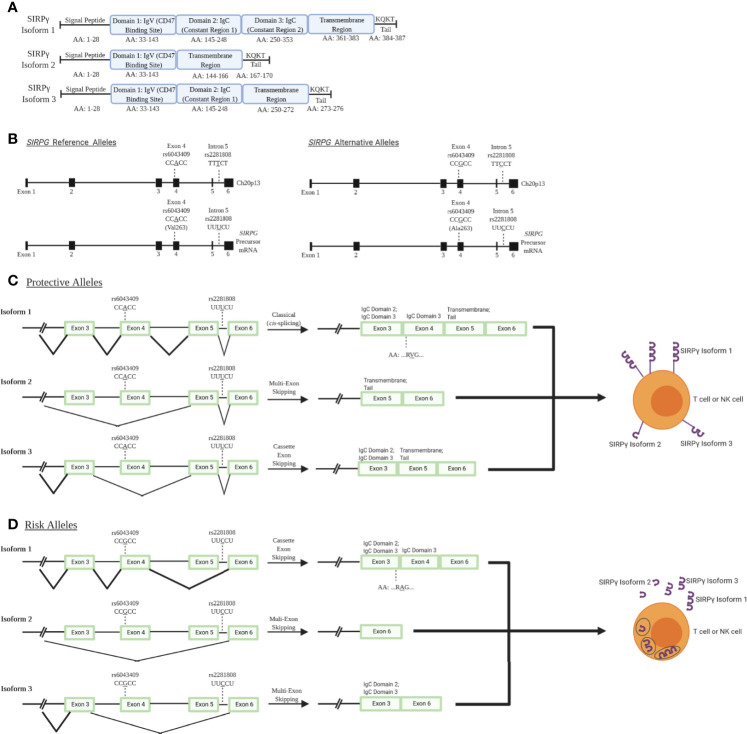
SIRPγ isoforms and predicted consequences of rs2281808 and rs6043409 SNPs: T1D-associated SNPs in signal regulatory protein gamma (*SIRPG*) may alter splicing activity and thereby surface SIRPG expression. **(A)** Isoform 1 (NCBI Reference Sequence: NP_061026.2) is the longest and most predominant form of the protein, while isoform 2 (NP_543006.2) and isoform 3 (NP_001034597.1) are shorter and less frequently observed (18, 19). All isoforms contain domain 1 (D1), which is the immunoglobulin variable (IgV) region that binds to CD47. However, only isoform 1 contains two immunoglobulin constant (IgC) regions and a known transmembrane region at the end of the protein structure. Isoform 3 contains at least one IgC, while isoform 2 has no constant region. **(B)** Gene and pre-mRNA diagrams of *SIRPG* (NCBI reference sequence for gene: NC_000020.11, Gene ID: 55423; precursor mRNA for isoform 1: NM_018556.41). Reference and alternative alleles for rs2281808 and rs6043409 are shown. **(C)** We speculate that the protective alleles of rs2281808 and rs6043409 are associated with “normal” *SIRPG* splicing and high membrane SIRPγ expression on T cells and NK cells, while **(D)** *SIRPG* risk alleles might promote aberrant splicing, potentially resulting in a loss of exon 5, which encodes most of the transmembrane region. We expect this would cause lower membrane expression of SIRPγ as well as increased SIRPγ secretion.

While much of the impact of *SIRPG* risk variants have yet to be empirically determined, bioinformatic analysis of the locus provides some insight into how variants may impact expression and processing. *SIRPG* is predicted to exhibit three different isoforms with isoform 1 being the most predominant and encoding the longest form of the protein. Alternative splicing results in the production of shorter isoforms 2 and 3 (modeled in **Figure 1A**) (6, 18, 19) which lack a majority of the transmembrane domain and thus, might allow for secretion of the SIRPγ protein rather than expression on the cell surface. The intronic T1D risk allele (C; rs2281808) may be associated with a decreased *SIRPG* intron-excision ratio in whole blood and spleen [Data Source: GTEx Analysis Release V8 (dbGaP Accession phs000424.v8.p2)] (20). Hence, we speculate that the risk allele could potentially increase the predominance of isoforms 2 and 3 due to interrupted splicing of the full-length isoform lowering overall SIRPγ expression on the cell surface (**Figures 1C, D**) (18, 19).

The exonic risk allele (G; rs6043409; alanine (Ala;A) codon) alters the structure of the extracellular D3 domain of SIRPγ, the function of which is currently unknown (**Figure 1B**) (15). It is possible that the conformation of the D1 and D2 domains of SIRPγ, which facilitate binding to the integrin-associated protein (IAP; CD47), could be impaired by the Ala mutation in the nearby D3 domain, inhibiting this protein-protein interaction (**Figures 1C, D**) (15). In turn, the protective allele [A; valine (Val;V) codon] could alter the D3 domain of SIRPγ and thereby enhance CD47 binding, but this has not been confirmed at this time (15). Further experiments are warranted to validate the predicted impacts of these risk variants on altered splicing and expression of *SIRPG*, along with their downstream effects on immune cell activation and function.

Although SIRPγ is the only member of the SIRP family with known T1D risk loci, other proteins found in this family, such as SIRPα and SIRPβ1, may also be involved in T1D pathogenesis. For example, the rs2281808 and rs6043409 risk variants are expression quantitative trait loci (eQTL) for both *SIRPG* and *SIRPB1*, whereby SIRPγ expression is reduced and SIRPβ1 expression is reciprocally increased (21). Thus, it is of importance to examine not only SIRPγ but also, other members of the SIRP family and their binding partner CD47 as a potential pathway of interest in T1D.

**Figure 2:**
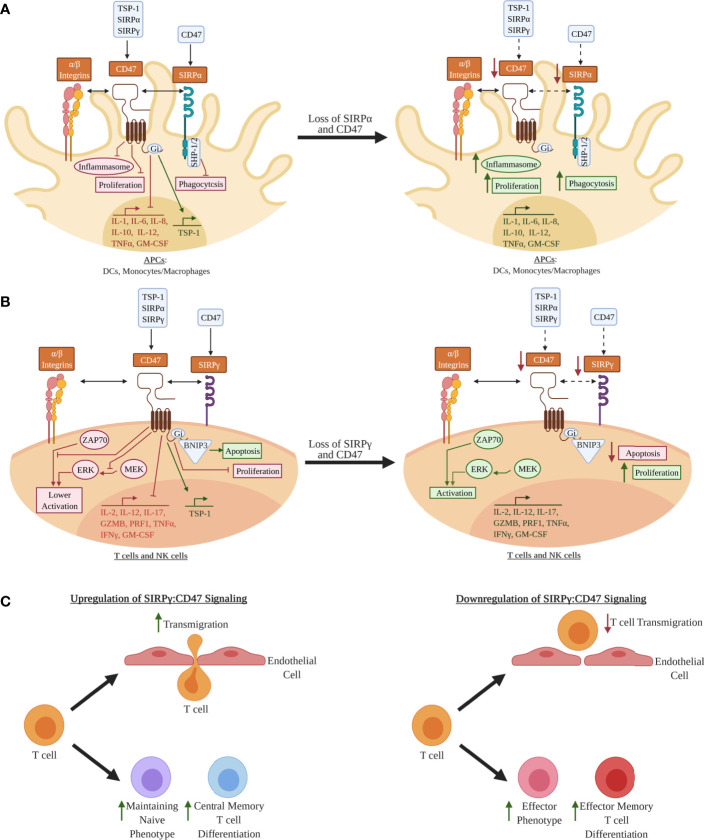
Hypothetical model for how decreased SIRPs CD47 signaling may lead to a pro-inflammatory phenotype in leukocytes: **(A)** In antigen-presenting cells (APCs) such as dendritic cells (DCs) and monocytes/macrophages, signal regulatory protein alpha (SIRPα) and CD47 are co-expressed, whereby SIRPα can bind CD47 expressed on other cell types (e.g., leukocytes, epithelial cells, endothelial cells) *in trans* or possibly *via in cis* interactions, thus activating the immunoreceptor tyrosine-based inhibitory motifs (ITIMs) on its cytoplasmic tail (5, 22, 23, 31, 32). CD47 is activated by either thrombospondin 1 (TSP-1), SIRPα, or SIRPγ, where it is hypothesized that inhibitory G protein (Gi) binding inhibits inflammasome activation, pro-inflammatory cytokine expression, proliferation, and phagocytosis (25, 33). These processes may be augmented if SIRPα and/or CD47 expression are decreased. **(B)** SIRPγ and CD47 are co-expressed in T and natural killer (NK) cells (5–7, 31). CD47 ligation is hypothesized to inhibit T and NK cell activation *via* inhibition of unknown downstream elements of the zeta chain of T cell receptor-associated protein kinase 70 (ZAP70) activation, inhibition phosphorylation of the mitogen-associated protein kinase (MEK), and inhibition of phosphorylation of the extracellular signal-regulated kinases (ERK) (26, 34–37). BCL2 interacting protein 3 (BNIP3) is a mediator of apoptosis that may be upregulated by activation of CD47 (24, 34, 38). Decrease expression of CD47 and/or SIRPγ could potentially augment T cells and NK cells in a way that results in a more activated phenotype and increase proliferation in these immune cells. **(C)** Other examples of the effect of SIRPγ:CD47 signaling in T cells. Upregulation of CD47 signaling is hypothesized to increase T cell transmigration; however, it is not known for sure if this increase in transmigration alters activation of the T cell (39, 40). CD47 ligation is also hypothesized to help maintain the naivety of T cells, and, once activated, promotes these T cells to differentiate to a more central memory phenotype (41–43). If loss of SIRPγ:CD47 signaling occurs in T cells, it is hypothesized that this will contribute towards the differentiation to more effector and effector memory phenotypes along with a loss of transmigration. Red Text/Box: Inhibition; Green Text/Box: Activation.

CD47 is ubiquitously expressed and is well known for providing a “don’t eat me” signal *via* binding to SIRPα on macrophages, which prevents macrophage-mediated phagocytosis and destruction of CD47-expressing target cells (22, 23). CD47 is also involved in the regulation of apoptosis, proliferation, adhesion, and migration of immune cells (24–28) as well as metabolic signaling in β-cells (29, 30). However, there remains limited understanding of the functional impact of *SIRPG* risk variants on SIRP:CD47 signaling and downstream immune cell activation and proliferation, as well as on pancreatic β-cell survival and function in the context of T1D pathogenesis. We hypothesize that the T1D-risk alleles of rs2281808 and rs604309 tagged to *SIRPG* might result in reduced CD47 binding capability or decreased expression of SIRPγ on T cells and NK cells (**Figure 2**). We pose that the SIRP:CD47 signaling pathway could be an important element in the regulation of autoimmunity. In this review, we describe the SIRP:CD47 signaling pathway and highlight potential functional implications of the T1D-associated *SIRPG* SNPs. We also discuss various strategies for modulating SIRPs/CD47 signaling to intervene in T1D.

## Structural Features and Signaling Pathways of SIRPs:CD47

### SIRP Family

SIRPα, SIRPβ1, and SIRPγ, which comprise the SIRP family, are type 1 transmembrane glycoproteins with three immunoglobulin-like (Ig-like) extracellular regions, a single transmembrane domain, and varying cytoplasmic domains (**Figure 2**) (5, 31). The cytoplasmic tail of SIRPα contains two immunoreceptor tyrosine-based inhibitory motifs (ITIMs), which interact with Src homology region 2-domain-containing phosphatase 1 (SHP1) and SHP2 (5, 31, 32). In antigen-presenting cells (APCs), such as dendritic cells (DCs) and macrophages/monocytes, SIRPα-induced SHP1/2 activation downregulates pro-inflammatory processes including cytokine/chemokine production, cellular adhesion, and phagocytosis (**Figure 2A**) (5, 31, 32). In contrast, SIRPβ1 and SIRPγ do not contain signaling motifs in their cytoplasmic domains. SIRPβ1 has a small six-amino acid tail that interacts with DNAX-activation protein 12 (DAP12), a transmembrane adaptor protein that contains immunoreceptor tyrosine-based activation motifs (ITAMs) (5, 31). SIRPγ, which has no ortholog in murine or other animal models, has a four-amino acid cytoplasmic tail that has not been shown to interact with adaptor proteins; thus, SIRPγ is hypothesized to function as a “decoy receptor” that competes for CD47 binding with SIRPα (5, 31). CD47 is a transmembrane protein in the Ig superfamily, with a single IgV-like domain at its extracellular N-terminus that binds to several integrins, vascular endothelial growth factor receptor-2 (VEGFR-2), CD36, Fas/CD95, thrombospondin-1 (TSP-1), SIRPα, and SIRPγ (23, 34). CD47 contains five membrane-spanning segments and a C-terminus cytoplasmic domain. Upon ligand binding to CD47, a heterotrimeric inhibitory G protein (Gi) is recruited to its cytoplasmic tail (25, 33), controlling various immunoregulatory processes, such as activation and apoptosis (through BCL2 interacting protein 3 (BNIP3) translocation) in DCs, monocytes/macrophages, T cells, and NK cells (**Figure 2A, B**) (24).

**Figure 3:**
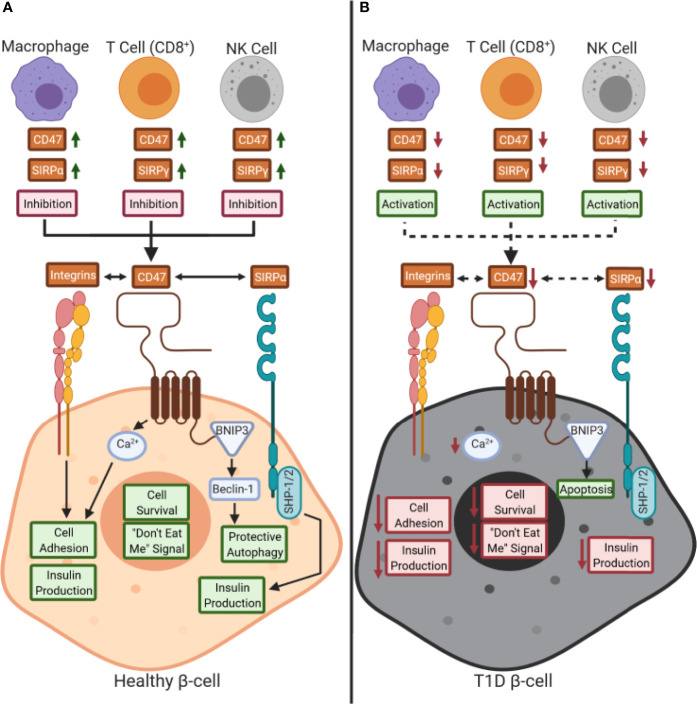
Working model of the role of CD47:SIRPs signaling in health and during type 1 diabetes pathogenesis: **(A)** CD47 and signal regulatory protein (SIRP) are expressed by immune cells during a healthy state. CD47 controls calcium (Ca^2+^) signaling in β-cells, through an unknown pathway, that promotes both cell adhesion and insulin production as well (29, 30, 34, 45). Also, CD47 is hypothesized to control protective autophagy *via* BCL2 interacting protein 3 (BNIP3) and Beclin-1 binding (34, 38). CD47 and SIRPα signaling can occur either *in trans* or *in cis* with each other or with other ligands, such as SIRPγ. We hypothesize that a reduction of SIRP expression and/or activity occurs on immune cells in type 1 diabetes (T1D), thus inhibiting peripheral immune tolerance. **(B)** Decreased CD47 and SIRPα activity in β-cells could potentially inhibit insulin secretion and cell survival while increasing immune cell-mediated destruction. Red text box: inhibition; green text box: activation; dashed arrows: inhibition.

SIRPα is expressed on a wide variety of cell types including many leukocyte subsets (e.g., monocytes, macrophages, DCs, NK cells), epithelial/endothelial cells, and other complex tissues (e.g., brain, pancreas) (31, 32, 44), while SIRPγ shows more restricted expression (**Figure 2A, B**) (5, 31). Specifically, SIRPγ is found on CD4^+^ and CD8^+^ T cells along with NK cells, where its function remains contested as promoting either activation or inhibition of these subsets (5, 31). SIRPβ1 is expressed on monocytes/macrophages and DCs, however, its ligand remains unknown as it does not bind to CD47 (5, 31, 32). Hence, this review will examine the SIRP:CD47 family signaling axis and its potential role in T1D pathogenesis, centering on the impacts of SIRPα, SIRPγ, and CD47 on immune cell function, along with that of SIRPα and CD47 on β-cell survival and insulin production (**Figure 3**).

### Implications of SIRPα:CD47 Signaling in APCs in T1D

The systematic failure to regulate self-antigen reactivity along with a pro-inflammatory cytokine signature has been shown to contribute to T cell-mediated destruction of β-cells in T1D (46, 47). Additionally, the involvement of APCs, such as monocytes/macrophages, in β-cell destruction has been hypothesized to further promote T1D pathogenesis (46–48). During pancreatic organogenesis, macrophage precursors are present in developing islets where they promote β-cell proliferation and survival (49–52). After islet maturation, β-cell mass increases during the postnatal period over the first two to three years of life, with tissue-resident macrophages playing a supporting role in growth and development (49–52). Throughout the pancreas, a tightly regulated balance of M1 (classically activated, pro-inflammatory) and M2 (alternatively activated, immunoregulatory) macrophage polarization occurs (49–52). M1 macrophages are required for protection from microbial infection and overall injury, whereas M2 macrophages are required to induce β-cell proliferation, cytotoxic protection, and prevent inflammatory responses (49–53). Throughout T1D pathogenesis, a significant increase in pancreatic tissue-resident M1 macrophages and a compensatory decrease in M2 macrophages occurs, increasing localized inflammation and promoting infiltration of more macrophages into the pancreas (46, 48, 54). As such, reduction of SIRPα and/or CD47 expression has been suggested to increase APC activation, proliferation, and phagocytic capacity (**Figure 2A**), characteristic of pro-inflammatory T1D-associated M1 macrophage polarization (31, 46–48, 54–56).

Once an immune response has been resolved and APC activation is no longer required, CD47 expression is upregulated to inhibit inflammasome activation (57–59) and the production of pro-inflammatory cytokines, including IL-12, TNF-α, IL-6, and GM-CSF (57, 58, 60). Inhibition of this pro-inflammatory milieu may contribute toward the mechanisms by which SIRPα modulates macrophage polarization. Accordingly, C57BL/6 mice with SIRPα overexpression exhibited an anti-inflammatory M2 macrophage phenotype while SIRPα knockdown promoted a predominantly pro-inflammatory M1 macrophage phenotype (61). M2 macrophages express higher levels of SIRPα than M1 macrophages, and M2 macrophage-secreted IL-8 has been shown to increase CD47 expression on disseminated colon cancer cells, thereby preventing their phagocytosis (62). Tseng and colleagues further demonstrated that treatment of the DLD1 human colon cancer cell line with anti-CD47 blocking antibody (clone B6H12) facilitated their phagocytosis by macrophages, which subsequently increased their ability to prime CD8^+^ T cells for proliferation and cytotoxicity as compared to macrophages cultured with DLD1 cells in the absence of anti-CD47 (63). Critically, a non-blocking anti-CD47 antibody (clone 2D3) did not impart comparable effects (63). Under the same conditions, B6H12 mediated phagocytosis of cancer cells reduced the ability of macrophages to stimulate CD4^+^ T cell proliferation but also, significantly reduced the percentage of CD4^+^FOXP3^+^ regulatory T cells in co-culture (63).

Similarly, during viral infections, CD47 expression increases on both immune cells and infected tissues due to an indirect effect of TNFα-NFκB1-signaling (64). It is hypothesized that this effect occurs to prevent the over-activation of immune cells during infection, but it remains unknown whether viruses or bacteria can directly influence CD47 expression to evade detection by the immune system (64). Regardless, once downregulation or blockade of SIRPα:CD47 signaling occurs, most immune cells exhibit enhanced anti-viral capabilities (64). In line with these findings, adoptively transferred CD47-deficient red blood cells (RBCs) are cleared more quickly than CD47^+^ RBCs in non-autoimmune C57BL/6 recipient mice, supporting the notion that CD47 expression is required for successful “don’t eat me” signaling (65). Interestingly, proinflammatory conditions or backcross to the autoimmune-prone non-obese diabetic (NOD) background (65) further accelerate the clearance of CD47-deficient RBCs (66). Altogether, these data suggest that SIRPα:CD47 signaling is particularly important for regulating immune responses in the context of cancer, infectious challenge and potentially, β-cell stress in subjects with high genetic risk for autoimmunity and specifically, T1D. Furthermore, these data suggest that interruptions or deficiencies in SIRPα:CD47 could promote the engulfment, processing, and aberrant presentation of self-antigens to T cells during the pathogenesis of T1D. Studies of SIRPα and/or CD47 expression on both DCs and monocytes/macrophages should be performed to elucidate the potential failure of this pathway in T1D pathogenesis.

### Implications of SIRPs:CD47 Signaling in T Cells and NK Cells in T1D

While autoreactive T cells are widely accepted as a key pathogenic feature of insulitis in organ donors with T1D (67, 68), the role of NK cells in T1D pathogenesis remains somewhat controversial (69). Although NK cells have been observed to infiltrate the human pancreas during T1D pathogenesis, NK cells are not required for disease onset in the NOD mouse model (69, 70). Nevertheless, we hypothesize that T1D-associated SNPs in the *SIRPG* locus contribute towards the decrease of SIRPγ expression on T cells and NK cells, potentially disrupting CD47 signaling and the downstream regulation that constrains the activation and proliferation of these subsets (**Figure 2B**). The impacts of T1D-associated *SIRPG* SNPs on T cell phenotype have been studied at the polyclonal level, but these observations must be validated in autoreactive islet-specific T cell clones (6, 7). SIRPγ^low^ CD8^+^ T cells isolated from healthy human donors demonstrated an effector gene signature characterized by increased *TBX21*, *EOMES*, *IFNG*, and *GZMB* expression, and possessed lower activation thresholds, determined through anti-CD3 titration *in vitro*, as compared to SIRPγ^high^ CD8^+^ T cells (6). These data support the hypothesis that decreased SIRPγ expression may enhance CD8^+^ T cell-mediated β-cell destruction in T1D.

The ligation of CD47 is hypothesized to inhibit T and NK cell activation *via* inhibition of unknown elements downstream of the zeta chain of T cell receptor (TCR)-associated protein kinase 70 (ZAP70) activation and subsequent phosphorylation of the extracellular signal-regulated kinases (ERK) from the mitogen-associated protein kinase (MAPK) signaling cascade (**Figure 2B**) (26, 34–37). Indeed, while phosphorylation of ZAP70 was unaffected in activated Jurkat T cell lines incubated with the CD47 ligand TSP-1, TSP-1 inhibited activation-induced expression of T cell early activation markers, such as CD69 and early growth response gene-1 (EGR-1), demonstrating that CD47 could be acting downstream of ZAP70 to inhibit T cell activation (35). Additional studies have demonstrated that activation of CD47 inhibits H_2_S signaling, which is a mediator of ERK signaling (26, 37). Thus, CD47 signaling is important in regulating T cell and potentially, NK cell activation through the MAPK pathway.

In contrast, however, one study found evidence that CD47 may instead promote the activation of T cells. Specifically, human Jurkat and primary human T cells or human CD47-transfected murine 3.L2 T cells stimulated by anti-CD3 and anti-CD47 activating antibodies, showed increased proliferation and IL-2 production as compared to those stimulated with anti-CD3 alone (71). Additionally, CD47 activation enhanced TCR zeta chain and ZAP70 phosphorylation (71). The cytoplasmic tail of CD47 was not necessary for these effects; rather, the membrane domain was required (71). The differing observations in this study (71) are thought to be due to activating antibodies eliciting a response from CD47 that contrasts from the quality or quantity of stimulation with TSP-1 in subsequent studies (26, 34–37). Thus, it is likely that endogenous CD47 signaling promotes T cell regulation; although, there are certainly unanswered questions regarding CD47 signaling in the context of other ligands.

Human tumor expression of CD47 has been shown to correlate with the expression of various co-inhibitory markers, such as program cell death protein 1 (PD-1) and cytotoxic T-lymphocyte associated protein 4 (CTLA-4), on tumor-infiltrating CD4^+^ and CD8^+^ T cells (72, 73). In mice, CD47 blockade contributed toward increased activation and cytotoxic potential of tumor-infiltrating CD8^+^ T cells (72, 73). Similarly, Seiffert et al. demonstrated that antibody-mediated blockade of either SIRPα or CD47 during DC priming of human CD8^+^ T cells reduced their anti-tumor cytotoxic activity *in vitro* (74). Disruption of SIRPα:CD47 signaling also increased NK cell activation and cytotoxicity while CD47 overexpression inhibited cytotoxic killing of tumor or MHC-deficient target cells *in vitro*; importantly, this latter observation was dependent upon SIRPα expression on NK cells (32). These mechanistic studies are crucial to inform novel therapeutic approaches capable of differentially targeting the SIRP:CD47 signaling pathway in cancer and autoimmune disease settings.

It has also been observed that CD47 signaling can control thymocyte selection, memory T cell differentiation, and CD4^+^ T helper (Th) cell skewing (25, 26, 41, 55). Intriguingly, Dugas, et al. observed that a *Cd47-*deficient transgenic mouse model, expressing an anti-hen egg lysozyme (HEL) TCR (clone 3A9) and HEL under the insulin promoter on the BALB/H-2k background, developed accelerated autoimmune diabetes concomitantly with a roughly two to four-fold decrease of regulatory CD4^-^CD8^-^ double negative (DN) T cells in the spleen and skin-draining lymph nodes compared to *Cd47*-sufficient controls (55). Briefly, DN T cells express a TCR but not CD4, CD8 or NK markers, and they exert antigen-specific negative regulation over effector T cells within peripheral blood (75). In the NOD model, DN T cells have been shown to prevent diabetes *via* production of IL-10 and/or cytotoxicity toward antigen-specific B cells (76, 77), regulatory mechanisms which may be lacking in the absence of CD47 expression. Meanwhile, defects in peripheral regulation may also be attributed to the loss of CD47-dependent T cell killing in the periphery (25).

Increased CD47 expression has been observed on naïve and central memory as compared to effector memory CD4^+^ T cells (**Figure 2C**) (41–43). CD47 expression is lower on activated than long-lived antigen-specific memory CD4^+^ T cells (41). When CD4^+^ T cells exhibit low CD47 expression, skewing shifts from a Th2 phenotype toward an inflammatory Th1 response (42). Together, these studies suggest that disruption of CD47 signaling could potentially augment T cell cytotoxicity and infiltration into pancreatic tissues while inhibiting regulatory T cell-mediated protection against β-cell destruction in T1D (**Figure 3**).

Interestingly, NOD mice carry a polymorphism in the *Sirpα* gene that induces an 18 amino acid variation in the IgV-like domain of the SIRPα protein, as compared to the non-obese diabetes resistant (NOR) strain (56). This variation was determined to increase the binding of SIRPα to CD47, thus increasing SIRPα:CD47 signaling between APCs and T cells (56). Furthermore, the authors showed that diabetogenic NOD.BDC2.5 CD4^+^ T cells exhibited increased proliferation and lower activation thresholds when co-cultured with NOD DCs with increased SIRPα binding, as compared to NOR DCs with decreased SIRPα binding to CD47 (56). More studies are required to understand how SIRPs:CD47 signaling affects priming and activation of CD4^+^ and CD8^+^ T cells, and the corresponding implications for T cell-mediated autoimmunity and T1D pathogenesis.

*SIRPG* does not have an orthologous counterpart in the mouse, limiting studies of this gene *in vivo*. In an *in vitro* co-culture model of human T cells and TNF-α activated human umbilical vein endothelial cell (HUVEC) monolayers under shear flow conditions, anti-CD47 and anti-SIRPγ antibodies prevented T cell transmigration across HUVECs (39, 40). Similarly, these authors observed a decrease in *in vitro* transendothelial migration of T cells across murine heart endothelial cell (MHEC) monolayers from CD47^-/-^ C57BL/6 mice in the presence of TNF-α induced inflammation (39, 40). Therefore, CD47 binding to SIRPα or SIRPγ can presumably alter T cell extravasation (**Figure 2C**). Whether SIRPγ:CD47 binding during transmigration is associated with modulation of T or NK cell activation remains of interest. These *in vitro* observations warrant further investigation of how SIRPγ:CD47 signaling might play a role in modifying cell migration using *ex vivo* platforms to study human pancreas (78–80) samples or humanized mouse models (81) xenografted with human cells to explore potential implications for islet infiltration and the development of insulitis in human T1D.

### Implications of SIRPα:CD47 Signaling in Pancreatic β-Cells in T1D

Previous literature examining the impact of SIRP:CD47 signaling has primarily focused on host immunity in the context of cancer development; hence, little is currently known about how this pathway relates to pancreatic β-cell development and survival in the context of T1D pathogenesis. CD47 forms clusters in lipid rafts on the surface of healthy cells, facilitating SIRPα ligation to inhibit phagocytosis by macrophages; in contrast, apoptotic cells exhibit a diffuse surface distribution of CD47 reducing the “don’t eat me” signal (82). Hence, in pancreatic β-cells, CD47 is hypothesized to promote survival *via* propagation of the “don’t eat me” signal in addition to regulating calcium (Ca^2+^) signaling associated with protection afforded by autophagy (**Figure 3**) (29, 30, 34, 45). CD47 promotes survival signaling through induction of the activator protein 1 (AP-1) transcriptional factor family, including the Jun (c-Jun, JunB, and Jun D) subset of transcription factors, in a majority of endothelial tissues and including β-cells (83–85). CD47 also enhances intracellular Ca^2+^ flux, which in terms of β-cell functionality, can contribute towards promoting cell adhesion along with triggering insulin secretion (86, 87). BNIP3 is bound to the cytoplasmic tail of CD47, where it can interact with Beclin-1, promoting protective autophagy through the unfolded protein response (UPR) which may occur in stressed β-cells (34, 38). Independent of immune-mediated destruction, SNPs tagged to genes involved in the regulation of apoptosis (protein tyrosine phosphatase non-receptor 2 (*PTPN2)*: rs1893217 and tumor necrosis factor, alpha-induced protein 3 (*TNFAIP3*): rs2327832) have been associated with T1D, with the risk variants resulting in increased apoptosis of β-cells (88, 89). Accordingly, we hypothesize that CD47 expression level or distribution may be altered on the surface of β-cells in individuals carrying risk alleles for these anti-apoptotic genes, thereby impacting β-cell survival.

Additionally, SIRPα is hypothesized to upregulate insulin secretion and/or production (**Figure 3**) (29, 31, 44, 54). Expression of SIRPα and CD47 colocalize with insulin staining in β-cells of C57BL/6 mice (29). High-fat diet-fed SIRPα^-/-^ mice exhibit reduced plasma insulin levels and impaired glucose tolerance as compared to wild-type mice, providing *in vivo* evidence that SIRPα can control insulin secretion in the context of metabolic stress (29). SIRPα phosphorylation is stimulated by insulin and insulin-like growth factor-1 (IGF-1); therefore, it is thought that SIRPα activation is controlled contemporaneously with insulin/IGF-1 receptor signaling (29, 90). This last observation is intriguing as we recently reported that IGF-1 levels are deficient before the clinical onset of T1D in at-risk subjects (91), potentially contributing to a decrease in SIRPα signaling in β-cells. The interaction between SIRPα and CD47, expressed on both APCs and pancreatic β-cells, might represent a key factor in T1D pathogenesis, and studies are warranted to examine their *in situ* expression in the human pancreas from control and T1D donors, impact on insulin production, and eQTL epistasis from single-cell sequencing data (92).

## Potential for SIRP:CD47-Modulating Therapeutics in T1D

Strategies focused on restoring or monitoring SIRPα, SIRPγ, and/or CD47 expression in subjects at-risk or with recent T1D onset may aid in the prediction, prevention or reversal of T1D. The *SIRPG* T1D-risk alleles and other T1D-risk loci have been associated with serological markers of disease progression. For example, a study conducted by The Environmental Determinants of Diabetes in the Young (TEDDY) consortium found that among individuals with the high-risk HLA-DR3/4 genotype, the minor (protective) allele for rs2281808 reduced the risk of islet autoantibody (AAb) seroconversion as compared to the major (risk) allele (93). These analyses suggest that *SIRPG* SNP genotypes may associate with high-risk HLA diplotypes, thus reinforcing the importance of examining the roles of both HLA and non-HLA risk SNPs in modulating events driving tissue-specific reactivity in the pathogenesis of T1D (93).

**Figure 4:**
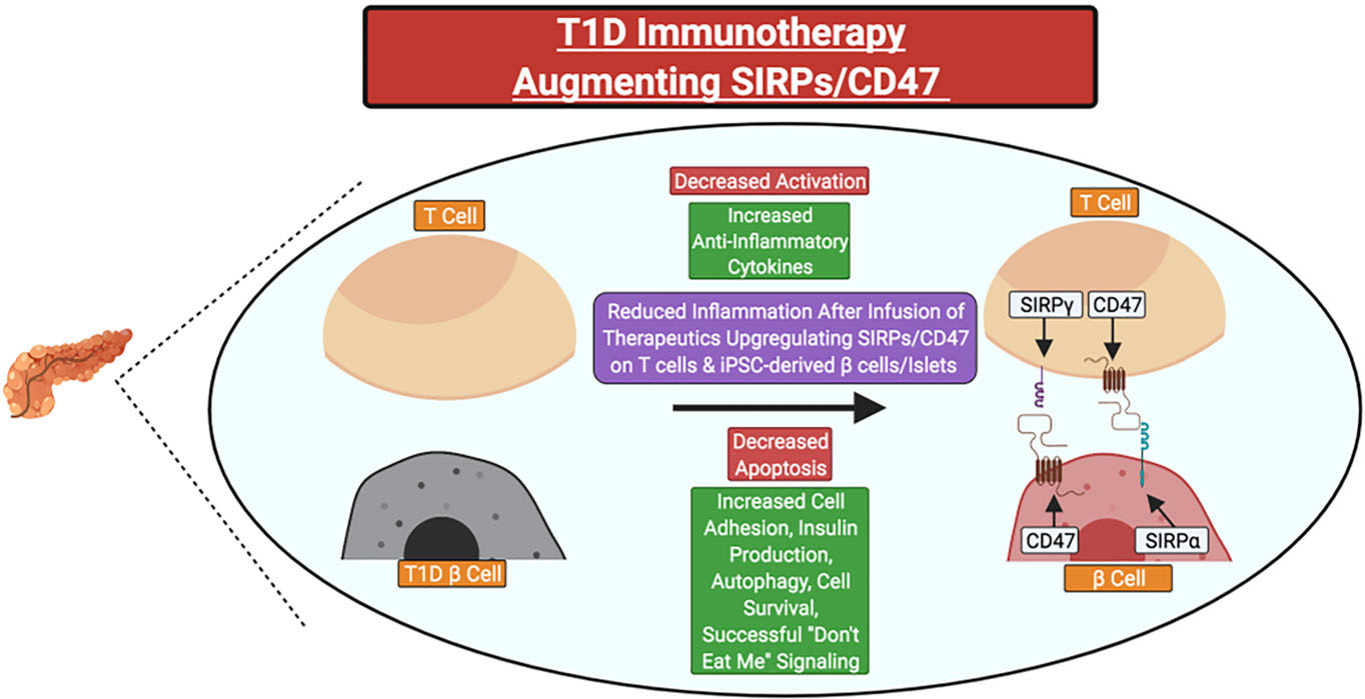
Potential clinical therapeutics targeting SIRPs and CD47 for type 1 diabetes prevention or suspension. Immunotherapies could be utilized to increase the expression of SIRPγ and/or CD47 on primary T cells for adoptive cell therapies or CD47 on stem cell-derived β-cells or islets before transplant to attenuate the magnitude of recurrent autoimmunity. Red Text Box: Expected Decrease; Green Text Box: Expected Increase.

In individuals with a family history of T1D or islet AAb seropositivity, *SIRPG* SNP genotyping could potentially allow for the identification of individuals who may benefit from SIRP:CD47 modulating therapies in precision medicine applications. Indeed, small molecule drugs or biologics that promote *SIRPG* expression or SIRPγ:CD47 signaling could serve as novel candidate therapies. Those that target CD47 (e.g., CD47 activating antibodies or SIRPγ:CD47 bi-specific antibodies) would likely be preferable for two key reasons: 1) SIRPγ:CD47 signaling occurs unilaterally downstream of CD47, and 2) the T1D-risk associated SNPs tagged to *SIRPG* are predicted to promote reduced SIRPγ expression and/or interaction with CD47 (5, 6, 20, 31, 35). Additionally, upregulation of CD47 and/or SIRPα expression on induced pluripotent stem cell-derived β-cells or islet transplants might augment their survival following β-cell replacement therapy in persons with longstanding T1D (**Figure 4**)

We hypothesize that SIRPγ expression on immune cells may be correlated with specific stages of T1D development in a manner governed by genetic risk variants in *SIRPG*, and thus, could be used as a biomarker of disease progression in conjunction with C-peptide and AAbs (12, 93, 94). In T1D subjects, *SIRPG* SNP genotype was associated with T1D risk at an early age (*P-value <0.05*, unadjusted), with the greatest effect at <7 years of age, an intermediate impact from 7 to 13 years of age, and a reduced impact at >13 years of age; therefore, therapeutic approaches involving *SIRPG* may have the highest efficacy at delaying or reducing T1D onset in younger patients (12, 93, 94).

## Discussion/Conclusion

As reviewed herein, prior research has suggested that SIRPs and CD47 could be involved in immunoregulation and cross-talk between immune cells as well as able to protect cells from targeted cellular destruction. However, it remains unclear how SIRP:CD47 signaling affects T cell activation in the periphery. We hypothesize that SIRP:CD47 represents a co-inhibitory pathway involved in immunoregulation. Because autoreactive T cells that bypass negative selection in the thymus are thought to express lower TCR affinities, SIRP:CD47 signaling may have an important effect on both central and peripheral tolerance during autoimmune disease pathogenesis. Therefore, we propose that novel immunotherapies that upregulate the expression of SIRPγ on T cells or increase CD47 signaling in persons with recent-onset or pre-T1D could ultimately serve as a powerful therapeutic approach to inhibit autoimmune destruction. However, additional research, including genotype/phenotype population studies, novel gene and SNP editing approaches, and longitudinal natural history studies are required to determine if the SIRP:CD47 signaling pathway could serve as an informative predictive biomarker of this disease or viable target for immune modulation.

## Author Contributions

RCS: Conceptualization, Investigation, Writing-Original Draft, Visualization. MEB: Conceptualization, Investigation, Writing-Original Draft, Visualization. MRS: Investigation, Writing-Review and Editing. ALP: Investigation, Writing-Review and Editing. TMB: Writing-Review and Editing, Supervision, Project Administration, Funding Acquisition. All authors contributed to the article and approved the submitted version.

## Funding

Efforts related to the content reviewed herein are supported by grants from the National Institutes of Health NIAID (P01 AI042288 to TMB), NIDDK Human Islet Research Network (HIRN; UG3 DK122638 to TMB), the University of Florida (UF) Experimental Pathology Innovation Grant (EPIG; 2908EPIG to RCS), HIRN Emerging Leaders in T1D (Human Islet Research Enhancement Center (HIREC) U24 DK104162 to RCS), and The Leona M. & Harry B. Helmsley Charitable Trust (Grant# 2018PG-T1D071 and Grant# 2004-03813 to TMB). Research related to this Review was also supported by the Network for Pancreatic Organ donors with Diabetes (nPOD; RRID : SCR_014541), a collaborative type 1 diabetes research project sponsored by JDRF (nPOD:5-SRA-2018-557-Q-R, 25-2013-268, 25-2012-380, and 25-2007-874 to MAA) and The Leona M. & Harry B. Helmsley Charitable Trust (Grant# 2018PG-T1D053).

## Conflict of Interest

The authors declare that the research was conducted in the absence of any commercial or financial relationships that could be construed as a potential conflict of interest.

## Publisher’s Note

All claims expressed in this article are solely those of the authors and do not necessarily represent those of their affiliated organizations, or those of the publisher, the editors and the reviewers. Any product that may be evaluated in this article, or claim that may be made by its manufacturer, is not guaranteed or endorsed by the publisher.

## Abbreviations

T1D, Type 1 Diabetes; HLA, Human Leukocyte Antigen; INS, Insulin; SIRP, Signal Regulatory Protein; APCs, Antigen-Presenting Cells; SNPs, Single Nucleotide Polymorphisms; NK, Natural Killer; MAF, Minor Allele Frequency; OR, Odds Ratio; LD, Linkage Disequilibrium; RA, Rheumatoid Arthritis; SLE, Systemic Lupus Erythematosus; UC, Ulcerative Colitis; IAP, Integrin-Associated Protein; eQTL, Expression Quantitative Trait Loci; Ig, Immunoglobulin; ITIMs, Immunoreceptor Tyrosine-based Inhibition Motifs; SHP 1/2, SH2-domain-containing Protein Phosphatase 1/2; DC, Dendritic Cells; DAP12, DNAX-Activation Protein 12; ITAMs, Immunoreceptor Tyrosine-based Activation Motifs; VEGFR-2, Vascular Endothelial Growth Factor Receptor-2; TSP-1, Thrombospondin-1; Gi, Inhibitory G Protein; BNIP3, BCL2 Interacting Protein 3; RBCs, Red Blood Cells; NOD, Non-Obese Diabetic; HUVEC, Human Umbilical Vein Endothelial Cell; PD-1, Program Cell Death Protein 1; CTLA-4, Cytotoxic T-Lymphocyte-Associated Protein 4; IFN-γ, Interferon Gamma; Th, T helper; HEL, Hen Egg Lysozyme; MHEC, Murine Heart Endothelial Cell; NOR, Nonobese T1D-resistant; FOXP3^+^, Forkhead box P3; TCR, T Cell Receptor; DN, Double Negative; ZAP70, Zeta-chain-Associated Protein; ERK, Extracellular signal-Regulated Kinases; MAPK, Mitogen-Associated Protein Kinase; H_2_S, Hydrogen Sulfide; EGR-1, Early Growth Response gene-1; PTPN2, Protein Tyrosine Phosphatase Non-receptor 2; TNFAIP3, Tumor Necrosis Factor, Alpha-Induced Protein 3; Ca^2+^, Calcium; AP-1, Activator Protein 1; UPR, Unfolded Protein Response; IGF-1, Insulin-Like Growth Factor-1; TEDDY, The Environmental Determinants of Diabetes in the Young.
